# Advanced Renal Failure in a Down Syndrome Patient due to Delayed Diagnosis of Non‐Neurogenic Neurogenic Bladder (Hinman Syndrome): A Case Report

**DOI:** 10.1002/ccr3.70879

**Published:** 2025-09-08

**Authors:** Ilkhan Rezaei, Fatemeh Rezaei

**Affiliations:** ^1^ Assistant Professor of Nephrology, Internal Medicine Department Kermanshah University of Medical Sciences Kermanshah Iran; ^2^ Clinical Research Development Center—Imam Reza Hospital—Kermanshah University of Medical Sciences (KUMS) Kermanshah Iran

**Keywords:** CKD, down syndrome, ESRD, Hinman syndrome

## Abstract

Nonneurogenic neurogenic bladder (NNB), also known as Hinman syndrome, is a rare medical condition characterized by clinical features resembling neurogenic bladder but without any neurological damage. This syndrome typically presents in children during the toilet‐training age but has also been reported in adults. It is more common among patients with Down syndrome regardless of age, likely due to their lower intellectual abilities. Early diagnosis of NNB leads to a favorable prognosis; however, delayed diagnosis and treatment can result in irreversible renal damage and even end‐stage renal disease (ESRD), as illustrated in this case. Obtaining accurate medical history from patients with Down syndrome is challenging because they may not report symptoms critical for early diagnosis. Therefore, clinicians should exercise caution when taking histories from this patient population.

## 1 Introduction

Nonneurogenic neurogenic bladder syndrome, also known as Hinman syndrome, is a rare disorder characterized by urinary bladder dysfunction with clinical and radiological features similar to those of neurogenic bladder but without any evidence of neurological damage [1]. This syndrome predominantly occurs in children, especially during the toilet‐training period, but has also been reported in adults [2]. Previous studies suggest that the incidence of this syndrome may be higher in patients with Down syndrome compared to the general population [3]. Advanced renal failure secondary to Hinman syndrome is uncommon and is usually reported only in cases of delayed diagnosis, particularly in developing countries [4].

In this article, we present an adult patient with Down syndrome who developed progressive renal failure due to delayed diagnosis of Hinman syndrome. To the best of our knowledge, this case of end‐stage renal failure is among the first reported in this age group worldwide [5].

## 2 Case History/Examination

A 39‐year‐old female was referred to the emergency department (ED) due to abnormal laboratory results indicating anemia, pancytopenia, and elevated serum blood urea nitrogen (BUN) and creatinine (Cr). She had initially presented to an internist complaining of bruising on her right forearm and thigh.

Her medical history was notable for Down syndrome and the aforementioned bruising. Further history revealed that the patient had significantly decreased urinary frequency in relation to her fluid intake, accompanied by episodes of large‐volume voiding. She denied symptoms such as weakness, lethargy, anorexia, dyspnea, and dysuria, and reported no prior history of urinary tract infections.

There was a family history of hematologic malignancy in her older brother (specific diagnosis unknown), who had died years earlier. No baseline creatinine measurements were available for comparison.

Physical examination revealed ecchymoses on the anterior aspect of the right forearm and thigh, with marked conjunctival pallor. No other abnormalities were noted.

## 3 Methods

Due to elevated serum urea (129 mg/dL) and creatinine (13.6 mg/dL), emergency hemodialysis was initiated. A Foley catheter was inserted, resulting in an initial urinary output of 2 L. Further investigations were undertaken to determine the cause of the renal impairment.

Vital signs were stable, and intravenous fluids (500 cc normal saline) were administered in the ED.

Due to the critically low hemoglobin level of 2.9 g/dL, the patient received an iso‐group and cross‐matched packed red blood cell (RBC) transfusion. However, about 30 min into the transfusion, she experienced back pain and shortness of breath. The transfusion was immediately stopped, and both the blood samples and the RBC unit were sent for laboratory testing. Laboratory evaluation revealed that the transfusion reaction resulted from incompatibility related to the patient's rare blood subgroup (C‐negative/Kell‐negative). Subsequently, compatible RBC units were transfused, resulting in a gradual increase in hemoglobin levels.

Iron studies and additional laboratory tests yielded the following results:

Reticulocyte count: 1.2%.

Serum iron: 169 μg/dL.

Ferritin: 49.9 ng/mL.

Direct Coombs test: Negative.

Indirect Coombs test: Negative.

Further laboratory results are summarized in Table 1.

**TABLE 1:** Laboratory data.

	Pre‐hospitalization	In ED	First day	Second day	5th day	8th day	Pre‐discharge
WBC	**3000**	3100	4100	3400	4700	4000	**6200**
RBC	**1.76**	1.6	4.68	2.27	3.61	4.47	**3.7**
Hb	**3.7**	2.9	3.2	4.8	9	11.4	**9.3**
Platlet	**90**	86	75	76	85	92	**83**
Urea	**242**	129	213	136	44	33	**22**
Cr	**12.69**	13.6	11.5	11.4	3.9	3.5	**2.8**
K	**6.5**	6.3	6.5	3.8	3.8	4.2	**4.3**
Ca	6.5	_	5.4	_	_	_	_
P	9.8	_	6.6	_	_	_	_
LDH	409	_	405	_	_	_	_

*Note:* The blood values presented in Table 1 are provided to illustrate the effects of the treatments administered, particularly hemodialysis, on the patient’s clinical status, with emphasis on renal function, anemia, and other consequences of renal failure.

Although hyperkalemia was detected, the electrocardiogram (ECG) was normal. Potassium levels normalized following intravenous fluid therapy.

Renal and urinary tract ultrasound demonstrated enlarged kidneys with severe bilateral hydronephrosis and renal cortical atrophy. The renal parenchyma appeared hyperechogenic with an irregular surface and evidence of scarring. The urinary bladder was markedly distended with diffuse wall thickening and remained severely distended even after voiding attempts.

**FIGURE 1:**
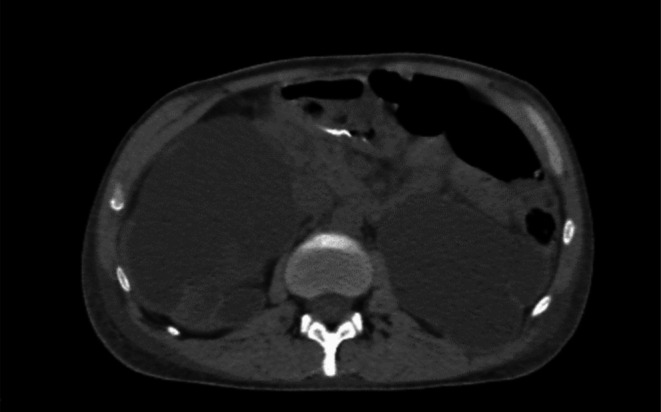
Abdominopelvic CT scan showing severe bilateral hydronephrosis.

**FIGURE 2:**
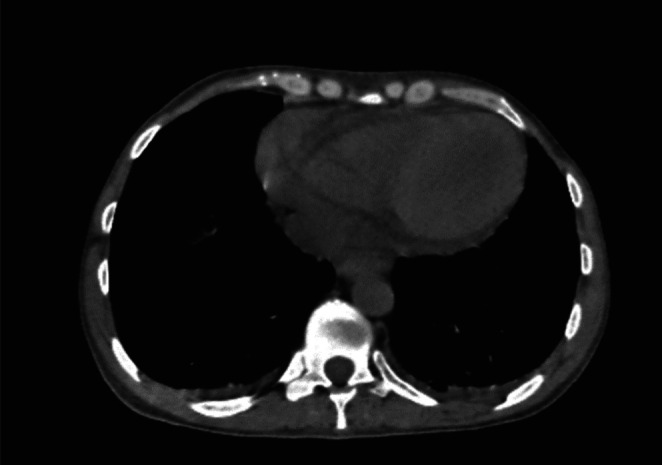
Chest CT scan showing mild bilateral pleural effusion and pericardial effusion (8 mm).

Non‐contrast abdominopelvic computed tomography (CT) confirmed severe bilateral hydronephrosis (Figure 1) with dilated renal pelves but normal ureteral caliber. No urinary obstruction was identified. Small amounts of free fluid were observed in the abdominal and pelvic cavities. Mild bilateral pleural and pericardial effusions (8 mm) were also noted (Figure 2).

Urodynamic studies revealed impaired bladder sensation and detrusor‐external sphincter dyssynergia. Lumbar spine magnetic resonance imaging (MRI) was performed to exclude neurological causes; no abnormalities were found.

Initially, urinary obstruction was suspected; however, no obstruction was found on imaging studies. Based on clinical history, examination, imaging, and absence of neurological or obstructive pathology, a diagnosis of Hinman syndrome (non‐neurogenic neurogenic bladder) was established.

The patient underwent six hemodialysis sessions via right femoral Shaldon catheter during hospitalization. An arteriovenous fistula (AVF) was created, and 1 day prior to discharge, a right clavicular catheter was placed for continued hemodialysis until AVF maturation.

No organomegaly or lymphadenopathy was detected on physical exam or imaging. Due to severe anemia, bone marrow biopsy or aspiration was not performed. Rheumatologic tests were negative.

## 4 Conclusion and Results

The patient was hospitalized for 8 days. Renal function did not improve, and she was classified as having chronic renal failure (CRF). Long‐term hemodialysis via AVF was planned alongside urological interventions. She was discharged in stable condition and referred to a nephrologist for ongoing management and to a urology clinic for bladder catheterization or clean intermittent catheterization, depending on clinical evaluation.

Due to a family history of hematologic malignancy, she was also referred to a hemato‐oncology clinic for further evaluation.

## 5 Discussion

Patients with Down syndrome typically have intellectual disabilities, which may hinder effective symptom reporting and delay diagnosis, leading to irreversible complications.

Nonneurogenic neurogenic bladder (NNB) has a favorable prognosis if treated early; however, delayed diagnosis can result in chronic renal failure. Given the increased risk of NNB and its complications in patients with Down syndrome, clinicians must be vigilant when taking medical histories, considering that patients may not be fully aware of their urinary symptoms. Early diagnosis and treatment are critical prognostic factors. Therefore, assessment of blood urea nitrogen (BUN) and creatinine, along with detailed history taking, can prevent irreversible renal damage.

## Author Contributions

**Ilkhan Rezaei:** validation, writing – review and editing. **Fatemeh Rezaei:** investigation, writing – original draft.

## Consent

Complete written informed consent was obtained from the patient's guardian for taking part in this study and publication of it.

## Conflicts of Interest

The authors declare no conflicts of interest.

## Data Availability

Research data are not shared.
